# Supplementary material for: Storing quantum coherence in a quantum dot nuclear spin ensemble for over 100 milliseconds
Source: Nat Commun. 2025 Dec 4;17:239. doi: 10.1038/s41467-025-66948-6 (PMC12783769; doi:10.1038/s41467-025-66948-6)
Supplement: Supplementary file 1 — Supplementary Information [file 41467_2025_66948_MOESM1_ESM.pdf]

Supplementary Information: Storing quantum coherence in a quantum dot nuclear spin ensemble for over 100 milliseconds

Harry E. Dyte

School of Mathematical and Physical Sciences,

University of Sheffield, Sheffield S3 7RH, United Kingdom

Santanu Manna, Saimon F. Covre da Silva, and Armando Rastelli

Institute of Semiconductor and Solid State Physics,

Johannes Kepler University Linz, Altenberger Str. 69, 4040 Linz, Austria

Evgeny A. Chekhovich

Department of Physics and Astronomy, University of Sussex, Brighton BN1 9QH, United Kingdom

(Dated: November 16, 2025)

Supplementary Note 1. SAMPLE STRUCTURE

The sample used for this work is the same as used in Refs. [1–4]. The sample is grown on a semi-insulating GaAs (001) substrate. Supplementary Fig. 1 shows the layer sequence of the semiconductor structure. Growth starts with a layer of $Al_{0.95}Ga_{0.05}As$ followed by a single pair of $Al_{0.2}Ga_{0.8}As$ and $Al_{0.95}Ga_{0.05}As$ layers, which act as a Bragg reflector in optical experiments. A 95 nm thick layer of $Al_{0.15}Ga_{0.85}As$ is then grown followed by a 95 nm thick layer of $Al_{0.15}Ga_{0.85}As$ doped with Si at a volume concentration of 1.0×10^{18} cm^{-3}. The concentration of Al is kept at a low value of 0.15 in the Si doped layer, in order to avoid the formation of the deep DX centers [5–7]. The n-type doped layer is followed by the electron tunnel barrier layers: first a 5 nm thick $Al_{0.15}Ga_{0.85}As$ layer is grown at a reduced temperature of 560 °C to suppress Si segregation, followed by a 10 nm thick $Al_{0.15}Ga_{0.85}As$ and then a 15 nm thick $Al_{0.33}Ga_{0.67}As$ layer grown at 600 °C. Droplets of Aluminium are grown on the surface of the $Al_{0.33}Ga_{0.67}As$ layer and are used to etch the nanoholes [8–10]. Atomic force microscopy shows typical nanoholes have a depth of $\approx$ 6.5 nm and are $\approx$ 70 nm in diameter at the top of the nanohole [1]. A 2.1 nm thick layer of GaAs is grown to form QDs by infilling the nanoholes and additionally form the quantum well (QW) layer. Thus, the maximum height of the QDs in the growth z direction is $\approx$ 9 nm. The GaAs layer is followed by a 268 nm thick $Al_{0.33}Ga_{0.67}As$ barrier layer. Finally, the p-type contact layers doped with C are grown: a 65 nm thick layer of $Al_{0.15}Ga_{0.85}As$ with a 5.0×10^{18} cm^{-3} doping concentration, a 5 nm thick layer of $Al_{0.15}Ga_{0.85}As$ with a 9.0×10^{18} cm^{-3} concentration,

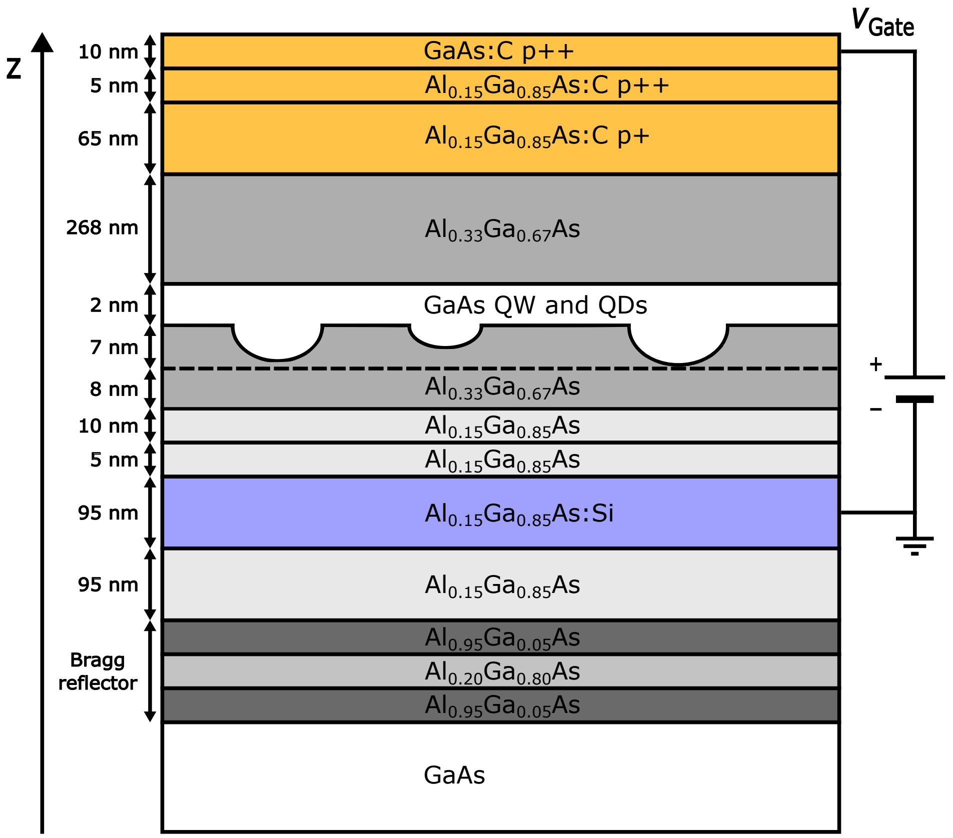

Supplementary Figure 1. Schematic of the quantum dot sample structure.

and a final 10 nm thick layer of GaAs with a 5.0×10^{18} cm^{-3} concentration.

Next, the sample is processed into a p-i-n diode structure. Mesa structures with a height of 250 nm are created by etching away the p-doped layers and depositing onto the etched regions the following sequence of layers: Ni(10 nm), AuGe(150 nm), Ni(40 nm), Au(100 nm). The sample is then annealed to enable diffusion of the deposited metals down to the n-doped layer to form the ohmic back contact. Depositing Ti(15 nm)/Au(100 nm) on to the p-type surface of the mesa areas forms the top gate contact. QD photoluminescence (PL) is excited and collected through the top of the sample. Sample gate bias V_{Gate} is the bias of the p-type top contact with respect to the grounded n-type back contact. Tunneling of holes is suppressed due to the large thickness of the top $Al_{0.33}Ga_{0.67}As$ layer, whereas tunnel coupling to the n-type layer enables deterministic charging of the QDs with electrons by changing V_{Gate}. For this work however, we leave the QD uncharged by applying reverse bias.

To allow the quadrupolar components of the nuclear magnetic resonance (NMR) spectra to be

resolved, the semiconductor sample used in this work is subjected to uniaxial mechanical stress. To this end, the semiconductor wafer is first cleaved into a small piece with a rectangular surface area of 0.7 mm×2.35 mm. The edges of the rectangular profile are aligned along the [110] and [1$\bar{1}$0] crystallographic directions. The sample thickness along the [001] growth direction is 0.35 nm. Thus, the sample is a parallelepiped. The sample is then inserted into a home-made stress cell. This is done in such a way that the two 0.7 mm×0.35 mm surfaces of the sample are contacted to the flat titanium surfaces of the stress cell bracket. Finally, a titanium screw is directed along the 2.35 mm long edge of the sample in order to apply compressive stress [4].

Supplementary Note 2. EXPERIMENTAL TECHNIQUES

The sample is placed in a bath-cryostat and cooled using liquid He to ≈ 4.2 K. An inbuilt superconducting coil is used to apply a static magnetic field B_z up to 8 T along the z-axis parallel to the sample growth direction [001] and the optical axis (Faraday geometry). Therefore, the applied mechanical stress is perpendicular to the field and optical axis. Optical measurements are conducted using a confocal microscopy configuration. An aspheric lens with a focal distance of 1.45 mm and NA = 0.58 is used as an objective for optical excitation of the QD and for collection of photoluminescence (PL). The excitation laser is focused into a spot with a diameter of ≈ 1 μm. A two-stage Czerny-Turner spectrometer is used to analyze the collected PL. The light is collimated and directed to a plane diffraction grating at each stage. Upon dispersion, the light is focused by a mirror with a 1 m focal length. After the spectrometer, a pair of achromatic lens doublets transfer the spectral image onto a charge-coupled device (CCD) photo-detector with a magnification of 3.75. The changes in the spectral splitting, determined from the PL spectra of a neutral exciton X^0, allow measurement of the hyperfine shifts $E_{\rm hf}$, which is proportional to the nuclear spin polarization degree (Fig. 2a of the main text). Both the pump and probe laser pulses are formed using mechanical shutters. One more mechanical shutter is used to block the input of the spectrometer during the optical pumping.

A. Optical pumping of nuclear spin polarization

Supplementary Fig. 2 shows the full timing diagram for the NMR measurements used in this work. In the first part of the measurement cycle dynamical nuclear spin polarization is created by optically pumping the QD with a tunable single-mode circularly polarized diode laser. This is a well

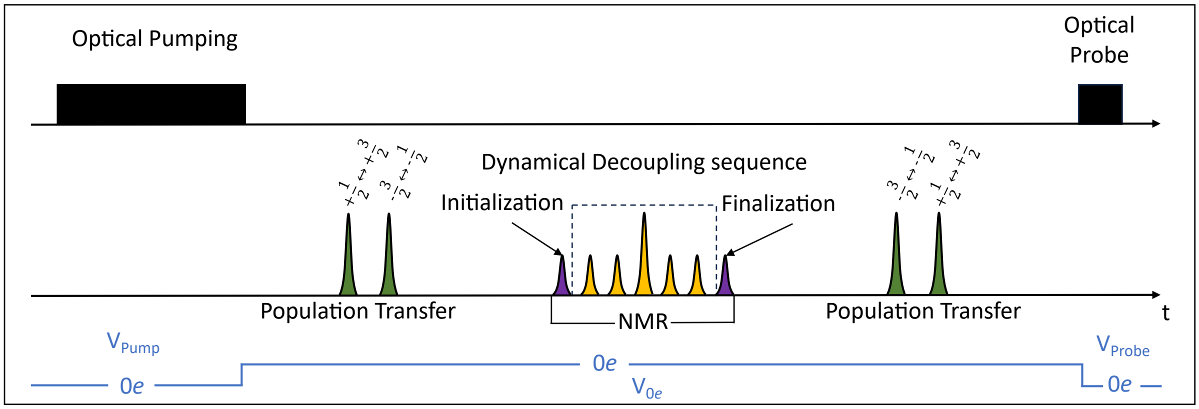

Supplementary Figure 2. Full timing diagram of pulsed dynamical decoupling experiments.

established technique and allows nuclear spin polarization greater than 50% to be reached [11–16]. The process is cyclic with three stages. First, optical excitation creates a spin polarized electron, a process enabled by the selection rules in III-V semiconductors allowing spin-polarized electron-hole pairs to be formed from the conversion of circularly polarized light. Secondly, the flip-flop term of the electron-nuclear hyperfine Hamiltonian allows the electron to exchange its spin with a single nuclei. Finally, electron-hole recombination removes the flipped electron allowing another polarized electron to be created in the dot and the process repeats, building-up polarization of the ensemble of nuclei. The pump laser pulse is typically 5 s long, afterwards, a 10 ms delay is added to ensure that the mechanical shutter has fully closed. The pump power ≈ 1 mW is three orders of magnitude greater than the ground-state PL saturation power, with typical photon energy $\approx 5-10$ meV above the X^0 PL energy. To ensure the QD is unoccupied during the pump process, a large reverse bias is applied $V_{\mathrm{Gate}} = -2.4$ V.

B. Nuclear magnetic resonance (NMR)

A copper wire coil is used to generate the magnetic field $B_x \perp z$ needed to carry out NMR experiments. The coil is positioned ≈ 0.5 mm from the QD sample. The coil is made of 10 turns of a 0.1 mm diameter enamelled copper wire wound on a ≈ 0.4 mm diameter spool in 5 layers, with 2 turns in each layer. A class-AB radiofrequency (Rf) amplifier (Tomco BT01000-AlphaSA rated up to 1000 W) is used to drive the coil, fed by the output of an arbitrary waveform generator (Keysight M8190).

The timing of NMR experiments proceeds as shown in Fig. 2. First, two Rf pulses are used to

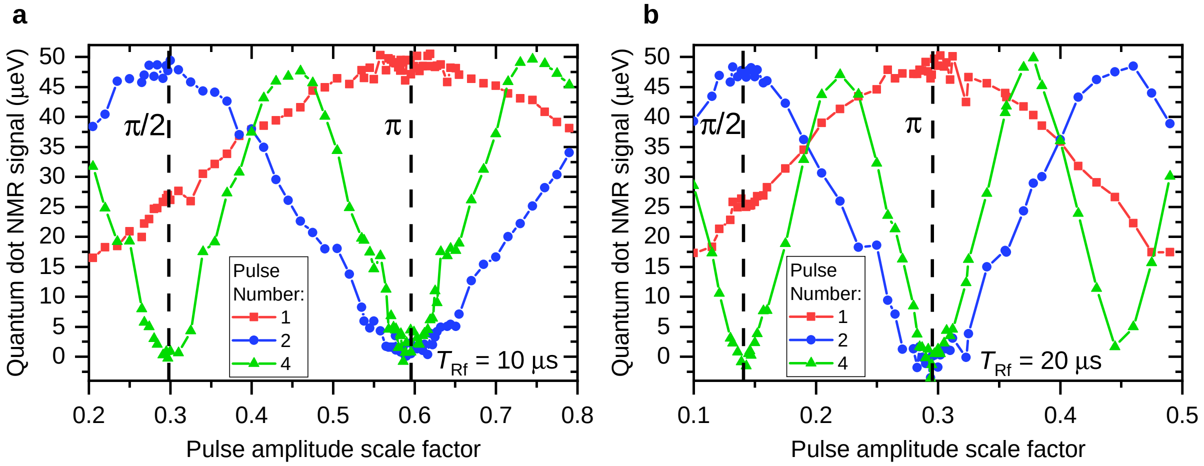

Supplementary Figure 3. **a** Rabi oscillations of the nuclear spins under Rf pulse bursts of increasing amplitude with fixed pulse duration $T_{\mathrm{Rf}} = 10\ \mu$s. Increasing the pulse amplitude increases the degree of rotation of the spins. The first peak in the oscillation (solid squares, red, one pulse) corresponds to a π rotation. We obtain the required amplitude of a $\pi/2$ rotation from the first peak for the oscillation driven by two pulses (solid circles, blue). Additionally we conduct measurements with 4, 8 and 16 pulses to improve the accuracy of the acquired amplitudes. Only the 4 pulse measurement (solid triangles, green) is shown for clarity. **b** Same as **a** but for pulses with $T_{\mathrm{Rf}} = 20\ \mu$s.

increase the NMR signal by transferring the nuclear spin population into the optimal configuration [4, 17]. Consider the case where the dot is pumped with σ^+ light leaving the $I_z = -3/2$ nuclear spin states as the most populated [3], while the $I_z = +3/2$ states are nearly unpopulated. By maximising the population change we maximize the NMR signal. For measurements performed on the central transition the population difference between the $I_z = -1/2$ and $I_z = +1/2$ states needs to be maximized. To this end we apply two π pulses to transfer the nuclear spin populations. First, a pulse is applied to the $+1/2 \leftrightarrow +3/2$ transition, exchanging their populations leaving $I_z = +1/2$ as the least populated state. Then, after a short delay, a second pulse is applied to the $-3/2 \leftrightarrow -1/2$ transition leaving $I_z = -1/2$ as the most populated state. A similar process can be applied in the case one of the satellite transitions is being investigated.

Next, the optically generated nuclear spin polarization needs to be converted from longitudinal polarization to transverse polarization. This is so the spins begin to decohere and the coherence time can be measured. We achieved this simply by using a single $\pi/2$ Rf pulse. The phase of the pulse can be varied to allow control over the initial orientation of the nuclear spins in the xy plane. Dynamical decoupling is then applied. The pulses for a given sequence are applied in a way that they are equally spaced by time τ, this means that while T_{FreeEvol} is increased the

time τ between each pair of adjacent pulses increases uniformly. Due to hardware limitations, the minimal interpulse delay achievable is $\tau \approx 0.3$ μs. This places a lower limit on the free evolution time that could be measured for a given dynamical decoupling sequence, based on the sequences number of pulses. Finally, a $\pi/2$ pulse is used to rotate the nuclei back along the z axis for optical readout. Again, the phase of finalization pulse can be varied. If the phase of the finalization pulse is the same as the initialization pulse, the nuclei are rotated such that their populations are antiparrallel compared to before the initialization pulse. Comparatively, if the finalization pulse is π out of phase with the initialization pulse, then the nuclear spins are aligned parallel to the orientation before initialization. All measurements of nuclear spin decoherence use initialization and finalization pulses with a π phase difference, as this allows us to avoid the range of nuclear polarizations where electron-nuclear bistability [18] accelerates the nuclear spin dynamics.

Before optical readout we again transfer the spin populations, this allows us to multiply the NMR signal by exploiting the entire $I = 3/2$ Hilbert space. The process is the reverse of the process described above. First, a π pulse is applied to the $-3/2 \leftrightarrow -1/2$ transition followed by another π pulse on the $+1/2 \leftrightarrow +3/2$ transition. The reason for this is that after application of the dynamical decoupling sequence, the remaining nuclear spin polarization is encoded in the $-1/2 \leftrightarrow +1/2$ states. Assuming a π phase difference between initial and final pulses, the $I_z = -1/2$ state will be most populated, while the $I_z = +1/2$ state will be least populated. The reverse population transfer means the $I_z = -3/2$ and $I_z = +3/2$ become the most and least populated states respectively, encoding the NMR signal into the $I_z = \pm 3/2$ subspace, approximately tripling the NMR signal measured optically. During NMR a bias of $V_{\rm Gate} = -1.5$ V is applied to the sample to ensure that there are no resident electrons.

C. Rf pulse calibration

Dynamical decoupling requires that precise rotations of the nuclei are performed. For some of the measurements in this work, as many as 2400 Rf pulses were applied to drive rotations of the nuclear spins, any inaccuracy in the rotations would therefore worsen the coherence of the nuclear spin polarization. To this end, we performed Rabi oscillation measurements to calibrate the π and $\pi/2$ Rf pulses. The Rf pulses are produced by modulating the Rf carrier with a raised cosine envelope function of total duration $T_{\rm Rf}$ between the two zero-amplitude points. For a desired $T_{\rm Rf}$, the amplitude of the applied pulses was increased and the resulting NMR signal measured, Supplementary Fig. 3a shows the Rabi oscillations observed for $T_{\rm Rf} = 10$ μs pulse duration. From

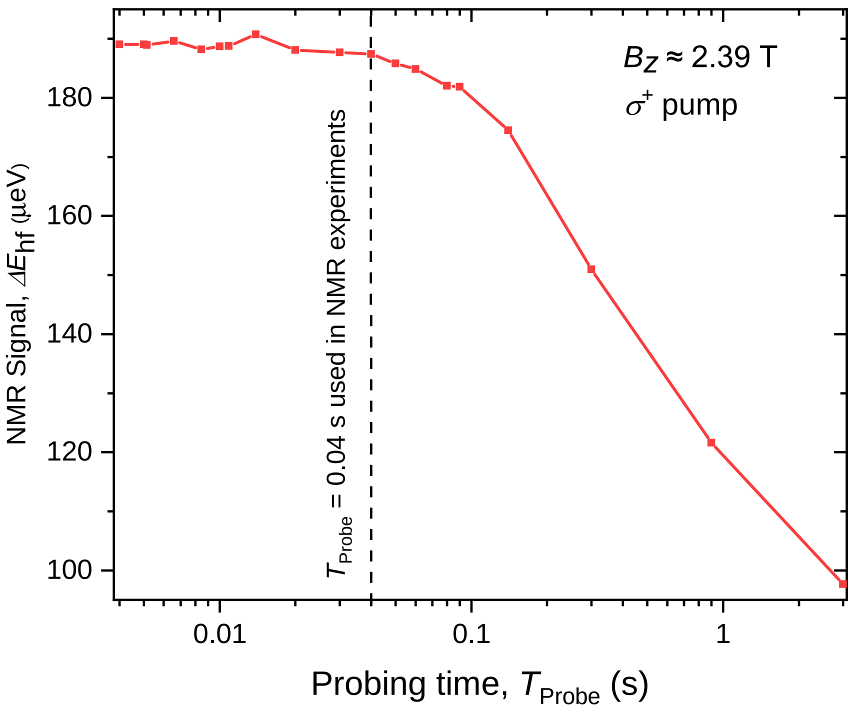

Supplementary Figure 4. Calibration measurement for the optical probe duration. Hyperfine shift measured under different duration optical probing after being polarized with σ^+ optical pumping. The time ($T_{\rm Probe}$) used for coherence measurements and nuclear spin decoupling is shown by the dashed line. Measurement was conducted with external magnetic field $B_{\rm z} = 2.39$ T.

this the power required for a π pulse is obtained by locating the first peak in the oscillation. The power needed for a $\pi/2$ Rf pulse is found by applying two separate pulses. Here, the first peak now gives the amplitude needed for a $\pi/2$ pulse, as this corresponds to a total rotation of nuclei by π meaning each of the two pulses will have rotated the nuclei by $\pi/2$. Finally, to improve the accuracy of these calibrations we conducted measurements with an increasing number of pulses: 4, 8, and 16. The Rabi oscillations were collectively fitted to obtain the power scaling factors. Supplementary Fig. 3b shows the same calibrations but for $T_{\rm Rf} = 20$ μs pulses.

D. CHASE dynamical decoupling sequences

Supplementary Table 1 defines the Rf pulses and the free evolution intervals of the CHASE-40 sequence cycle. Definitions of the shorter sequences CHASE-5, CHASE-10, and CHASE-20 that were introduced previously [19] are also provided.

Supplementary Table 1. Definition of the CHASE pulse sequences. The cycle of each sequence is given in curly brackets where x, y (−x, −y) represent positive (negative) $\pi/2$ rotations around the respective axes of the rotating frame, x^2, y^2, $-x^2$, $-y^2$ stand for the π rotations, while τ and 2τ are the free evolution intervals.

Sequence type	Sequence cycle definition
CHASE-5	$\{\tau,-x,\tau,y,\tau,x^2,\tau,y,\tau,x,\tau\}$
CHASE-10	$\{\tau,-x,\tau,y,\tau,x^2,\tau,y,\tau,x,2\tau,x,\tau,-y,\tau,-x^2,\tau,-y,\tau,-x,\tau\}$
CHASE-20	$\{\tau,-x,\tau,y,\tau,x^2,\tau,y,\tau,x,2\tau,x,\tau,-y,\tau,-x^2,\tau,-y,\tau,-x,2\tau,$ $x,\tau,y,\tau,x^2,\tau,y,\tau,-x,2\tau,-x,\tau,-y,\tau,-x^2,\tau,-y,\tau,x,\tau\}$
CHASE-40	$\{\tau,-x,\tau,y,\tau,x^2,\tau,y,\tau,x,2\tau,x,\tau,-y,\tau,-x^2,\tau,-y,\tau,-x,2\tau,$ $-y,\tau,-x,\tau,y^2,\tau,-x,\tau,y,2\tau,y,\tau,x,\tau,-y^2,\tau,x,\tau,-y,2\tau,$ $x,\tau,-y,\tau,-x^2,\tau,-y,\tau,-x,2\tau,-x,\tau,y,\tau,x^2,\tau,y,\tau,x,2\tau,$ $y,\tau,x,\tau,-y^2,\tau,x,\tau,-y,2\tau,-y,\tau,-x,\tau,y^2,\tau,-x,\tau,y,\tau\}$

E. Optical probing of nuclear spins

Measurement of the nuclear spin polarization is conducted using optical probing. The probe power and the bias during the probe ($V_{\mathrm{Gate}} = +0.9$ V) are chosen to maximize (saturate) the intensity of the ground neutral exciton (X^0) state PL. There is a 10 ms delay between the bias switching and the mechanical shutter activating, forming the probe pulse. This delay ensures that the charge state of the quantum dot is in its steady state when the optical probe pulse is applied. An example PL spectra is shown in Fig. 2a of the main text. The PL is generated by recombination of the electron-hole pairs. Under optical excitation the spin of the electron is random, meaning light is emitted from both bright exciton spin states. The PL is accumulated over millisecond timescales allowing both exciton states to be observed in the same PL spectrum. The applied magnetic field splits the two PL lines, these can be further shifted by the nuclear polarization via the hyperfine interaction. The shifts can be seen when the nuclei are polarized with different circularly polarized light. Injection of optically excited electrons into the dot leads to spin flip-flops between the electron spin and nuclei which will progressively depolarize the nuclei. A probe pulse duration measurement is shown in Supplementary Fig. 4 for external magnetic field of $B_{\mathrm{z}} = 2.39$ T. As the probe time is increased the nuclear polarization decreases, seen in the reduction of PL splitting. Similar results are observed in a wide range of magnetic fields $B_{\mathrm{z}} = 1 - 8$ T. Based on such calibration results we use a probe time of $T_{\mathrm{Probe}} = 0.04$ s, maximizing the collected signal from the optical excitation while reducing the loss in nuclear spin polarization due to the optically-induced

spin flip-flops. The rate of the probe-induced loss in nuclear spin polarization itself depends on the instantaneous value of the nuclear polarization (through electron-nuclear spin feedback [18]). This nonlinear dependence can result in distortion of the measured nuclear spin polarization. An example can be found in Fig. 2b of the main text, where the nuclear spin Rabi oscillations are seen to deviate slightly from an ideal sine-wave shape. The distortion is particularly notable in Fig. 2b due to the large range of the signal ($\approx 45\ \mu$eV). The decoherence measurements (e.g. Fig. 2c) are less affected by such nonlinear distortions due to the smaller signal range.

Supplementary Note 3. ADDITIONAL EXPERIMENTAL RESULTS

A. Optimal Rf pulse duration in dynamical decoupling

Ideally, nuclear spin rotations would be carried out instantaneously, minimising decoherence due to unwanted evolution of the nuclear spin ensemble during the rotation. As we are limited to using finite time Rf pulses, one may expect that shorter $T_{\rm Rf}$ times would yield better coherence storage performance. Yet, as discussed in the main text, we find that when using short $T_{\rm Rf} = 10\ \mu$s pulses we see a drop in performance for high pulse number sequences (≈ 640 pulses) compared to when longer $T_{\rm Rf} = 20\ \mu$s pulses are used. Here we present experimental results that reveal these counterintuitive observations. Nuclear spin decoherence measurements, such as shown in Fig. 2c of the main text, have been conducted both with $T_{\rm Rf} = 10\ \mu$s and $T_{\rm Rf} = 20\ \mu$s on the $I_{\rm z} = \pm 1/2$ nuclear spin subspace. In each such measurement, the total free evolution time $T_{\rm FreeEvol}$ is varied while keeping a constant number of pulses in the dynamical decoupling sequence. The resulting dependence of the spin echo NMR signal is fitted with a stretched exponential function of $T_{\rm FreeEvol}$. From the fit we derive the NMR signal in the limit of short free evolution $T_{\rm FreeEvol} \rightarrow 0$ and the characteristic nuclear spin coherence time T_2. These results are shown in Figs. 5a and b, respectively. The values are shown as a function of the total number of pulses in Hahn echo (1 pulse), CHASE-5 (5 pulses), CHASE-10 (10 pulses), CHASE-20 (20 pulses), and a varying number of CHASE-40 cycles (all points with ≥ 40 pulses). The results are shown for two different orientations of the initial nuclear spin polarization in the equatorial plane of the rotating frame (initialization pulse phase $\phi = 0$ and $\phi = \pi/2$).

From Supplementary Fig. 5a we find that for short pulse sequences ($\lesssim 100$ pulses) the short-evolution NMR signal is somewhat better (larger) for short pulses ($T_{\rm Rf} = 10\ \mu$s, squares), as these are better approximation to instantaneous spin Hamiltonian transformations than the $T_{\rm Rf} =$

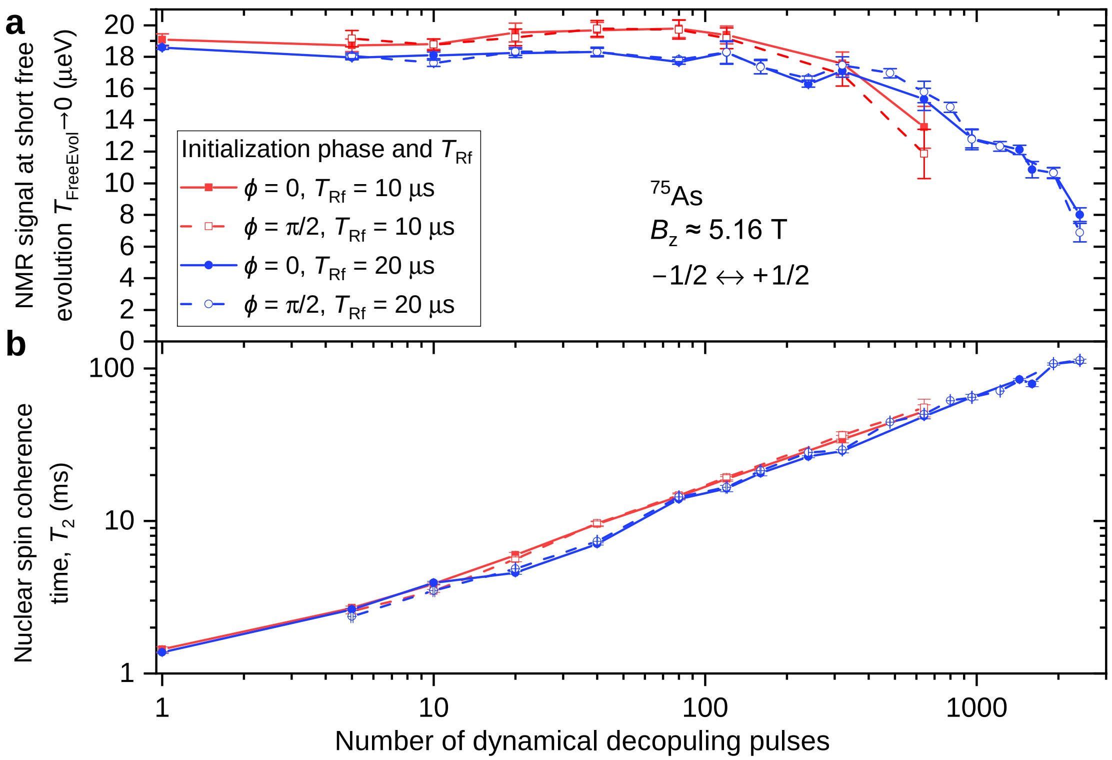

Supplementary Figure 5. **a** NMR signal (spin echo amplitude) at short free evolution time $T_{\mathrm{FreeEvol}} \to 0$ plotted as a function of the number of Rf pulses in the dynamical decoupling sequence. The data is shown for Rf pulse durations of $T_{\mathrm{Rf}} = 10$ μs (squares) and $T_{\mathrm{Rf}} = 20$ μs (circles). Measurements are conducted with initial nuclear polarization along the $-y$ axis (solid symbols, initialization Rf pulse phase $\phi = 0$) and the x axis ($\phi = \pi/2$, open symbols) of the rotating frame. **b** Dependence of the nuclear spin coherence time T_2 on the number of Rf pulses. All error bars are 95% confidence intervals.

20 μs pulses (circles) pulses. However, for longer sequences (640 pulses) the short-evolution spin echo under short pulses ($T_{\mathrm{Rf}} = 10$ μs) becomes worse than under long pulses ($T_{\mathrm{Rf}} = 20$ μs). Supplementary Fig. 5b shows that the coherence time T_2, which is a measure of decoherence during free evolution, is nearly independent of the Rf pulse duration T_{Rf}. This allows us to conclude that the reduced short-evolution NMR signal (i.e. reduced fidelity of coherence storage) at $T_{\mathrm{Rf}} = 10$ μs is a result of undesired spin evolution during the pulses, rather than pure decoherence during free evolution between the pulses. In particular, we attribute the reduced performance of the $T_{\mathrm{Rf}} = 10$ μs pulses to parasitic leakage of the stored state to the two nuclear satellite transitions, owing to the broader spectral profile of the pulses, as shown by the dashed lines in Fig. 1a of the main text.

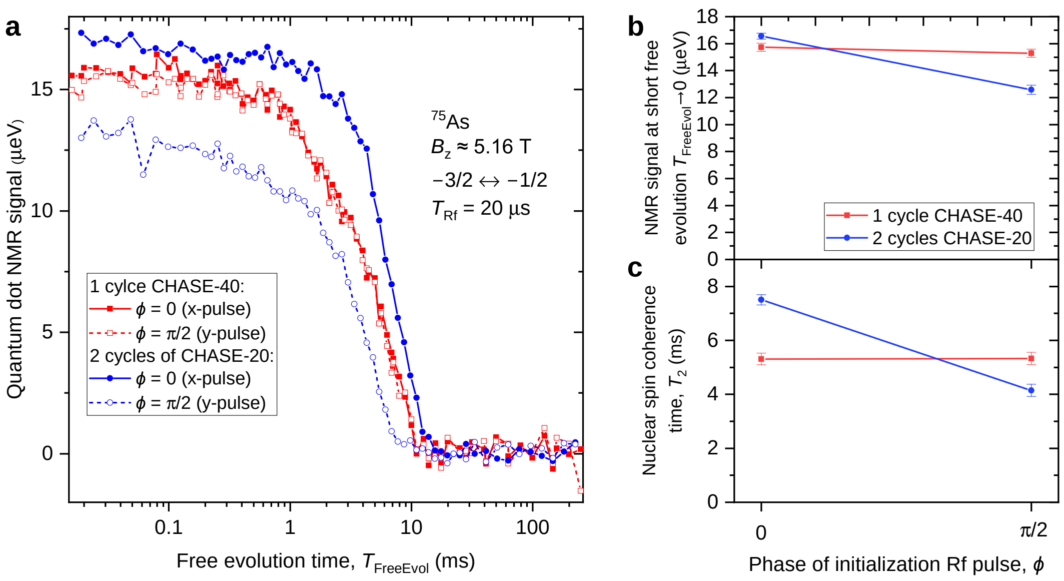

Supplementary Figure 6. **a** Decoherence measured on the $I_z = (-3/2, -1/2)$ nuclear spin subspace. Two cycles of CHASE-20 (squares) are compared to a single CHASE-40 cycle (circles) both when initializing the nuclear spin polarization along the $-y$ axis of the rotating frame (solid symbols, $\phi = 0$ Rf pulse phase) and along the x axis (open symbols, $\phi = \pi/2$). **b** NMR signal (spin echo amplitude) at short free evolution time $T_{\mathrm{FreeEvol}} \to 0$ plotted as a function of the phase ϕ of the initialization Rf pulse for two cycles of CHASE-20 (squares) and one cycle of CHASE-40 (circles). The values are obtained from fitting the decay curves in (**a**). **c** Same as (**b**) but for nuclear spin decoherence time T_2. All error bars are 95% confidence intervals.

B. Comparison of pulsed spin locking in CHASE-20 and CHASE-40 decoupling sequences

Pulsed spin locking is observed in various dynamical decoupling techniques. The simplest example is the Carr-Purcell sequence, where a train of π Rf pulses of the same phase is applied. The spin locking can be understood intuitively as an effective transverse magnetic field in the rotating frame that arises from repeated rotations of the nuclear spins around a preferential equatorial axis of the rotating frame [20]. If the coherent nuclear polarization, created by the initial $\pi/2$ Rf pulse, is parallel to this effective transverse magnetic field, the nuclear state becomes "locked" and its decay is suppressed. In other words, the transverse (T_2) coherence decay is replaced by a decay that is akin to longitudinal (T_1) relaxation in the rotating frame. By contrast, the initial state polarized orthogonal to the effective locking field undergoes precession and accelerated decoherence. In the case of the CHASE-10 cycle, shown in Fig. 1b of the main text, the two π rotations (labeled $+\mathrm{x}^2$ and $-\mathrm{x}^2$) create a preferential direction in the rotating frame, whereas the four $\pi/2$ rotation pulses labeled as $\pm$x are balanced by four $\pm$y Rf pulses rotating the spins around the orthogonal axis in

the rotating frame.

The CHASE-40 sequence is designed to be resilient against pulsed spin locking, by combining four CHASE-10 subsequences, where the phase of all Rf pulses is stepped in each block by $\pi/2$, as shown in Fig. 1c of the main text. Here we perform direct comparison of CHASE-40 with a sequences of pulses, where such phase stepping is omitted. To this end we use two cycles of CHASE-20 sequences, where each CHASE-20 block is a combination of CHASE-10 and its mirror copy, where all Rf pulses are applied in reverse [19]. Thus, both the CHASE-40 and 2 cycles of CHASE-20 differ only by the phases of the Rf pulses. In the case of CHASE-20, all the π rotations ($+\mathrm{x}^2$ and $-\mathrm{x}^2$) are around the same equatorial axis of the rotating frame.

The experiments are performed on the $I_z = (-3/2, -1/2)$ subspace of the ^{75}As nuclear spins. The resulting decays of nuclear spin echo with the increasing free evolution time T_{FreeEvol} are shown in Supplementary Fig. 6a. The decay curves are fitted with stretched exponentials to derive the NMR signal in the limit of short free evolution $T_{\mathrm{FreeEvol}} \to 0$ (shown in Supplementary Fig. 6b) and the characteristic nuclear spin coherence time T_2 (shown in Supplementary Fig. 6c). In the case of CHASE-40 (squares in Supplementary Fig. 6a) the decoherence (characterized by $T_2 \approx 5.3$ ms) does not depend on the direction of the initial transverse nuclear spin polarization. This is also in agreement with the data of Fig. 4 of the main text obtained for the $I_z = -1/2, +1/2$ nuclear spin subspace. These results confirm that CHASE-40 effectively eliminates the pulsed spin locking.

For the two cycles of CHASE-20 (circles in Supplementary Fig. 6a) we observe a pronounced difference in decoherence, depending on the initial nuclear spin state. For the spin state polarized along the $-y$ axis of the rotating frame (solid circles, $\phi = 0$) the decay is slowed down ($T_2 \approx 7.5$ ms), revealing the pulsed spin locking effect. (With the convention used here, the pulse labeled as "+x" in the sequence definition of Figs. 1b, c of the main text corresponds to $\phi = 0$. This pulse produces Rf magnetic field along the x axis of the rotating frame and initializes nuclear polarization along the $-y$ axis of the rotating frame. For the "−x" pulse the Rf phase is $\phi = \pi$. The "+y" pulse corresponds to $\phi = \pi/2$ and initializes the nuclear spin state along the x axis. The $\pm\mathrm{x}^2$ Rf pulses have the same phase as ±x, and so on).

For the case of CHASE-20 decoupling with an initial nuclear spin polarization along the x axis ($\phi = 0$), there is a pronounced reduction both in the nuclear spin echo amplitude at short free evolution $T_{\mathrm{FreeEvol}} \to 0$ and in $T_2 \approx 4.1$ ms, characterizing decoherence during free evolution. In the context of quantum memories, this can be interpreted as the worst case scenario, which is an inevitable side effect of the pulsed spin locking. This is why elimination of spin locking, such as achieved with CHASE-40, is important for operating nuclear spins as a quantum memory, which

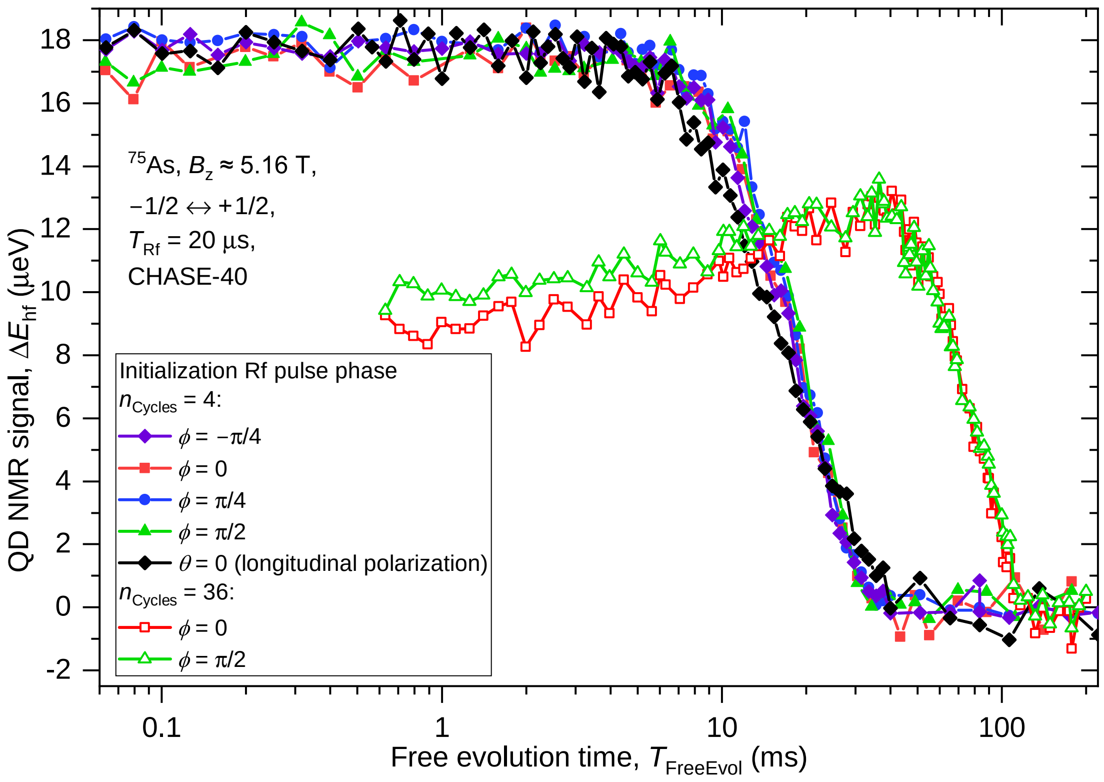

Supplementary Figure 7. **Uniform decoupling of an arbitrary coherent nuclear spin state.** Nuclear spin decoherence measured under CHASE-40 decoupling with different phases ϕ of the initialization Rf pulse, which initializes transverse nuclear polarization along different azimuth angles in the xy plane ($\theta = \pi/2$). The measurement of the longitudinal nuclear spin relaxation under CHASE-40 (without the initialization pulse, $\theta = 0$) is shown by the open diamonds. Results are shown for $n_{\mathrm{Cycles}} = 4$ cycles of the CHASE-40 sequence (solid symbols) and for $n_{\mathrm{Cycles}} = 36$ cycles (open symbols).

is generally expected to preserve an arbitrary coherent state.

C. Uniform decoupling of an arbitrary coherent nuclear spin state

Supplementary Fig. 7 shows an extended version of Fig. 4a of the main text. In addition to reproducing the data with $n_{\mathrm{Cycles}} = 4$ we show here the results measured with $n_{\mathrm{Cycles}} = 36$ cycles. The finite duration of the Rf control pulses ($T_{\mathrm{Rf}} = 20$ μs) and the large number of cycles ($n_{\mathrm{Cycles}} = 36$) leads to a substantial loss of coherence (approximately by a half) even in the limit of short free evolution ($T_{\mathrm{FreeEvol}} \rightarrow 0$). This decoherence during the Rf control pulses is seen to depend slightly on the direction of the initial transverse spin polarization. However, this type of decoherence can be eliminated in principle by reducing the duration of the Rf pulses (currently

limited by the magnitude of the strain-induced quadrupolar shifts). The fundamental properties of the decoupling sequence itself, i.e. the time order of the Hamiltonian states in the toggling frame of reference, are better characterised by decoherence during the free evolution intervals between the control pulses. We find that $n_{\mathrm{Cycles}} = 36$ cycles of CHASE-40 still result in very similar decoherence times ($T_2^{36\mathrm{xCHASE-40}} \approx 84$ ms) when measured with $\phi = 0$ and $\phi = \pi/2$ phases of the initialization Rf pulse. This corroborates our conclusion about uniform (isotropic) decoupling of an arbitrary coherent spin state, showing that it holds even under a large number of CHASE-40 cycles. The finite-pulse effects are significantly smaller at $n_{\mathrm{Cycles}} = 4$, providing a clearer picture of the uniform decoupling, as presented in the main text.

D. Details on Measurement of Nuclear Spin Transitions

The NMR Spectra of the ^{75}As nuclei used in Fig. 1a of the main text were obtained using two separate measurement techniques. For the $-1/2 \leftrightarrow +1/2$ central transition (blue in Fig. 1a) the spectrum was obtained from a Fourier transform of the measured free induction decay. The free induction decay was measured using $T_{\mathrm{Rf}} = 10\ \mu s$. The $-3/2 \leftrightarrow -1/2$ and $+1/2 \leftrightarrow +3/2$ satellite transitions (black in Fig. 1a) were measured using the inverse NMR measurement technique [21].

Supplementary Note 4. DISCUSSION OF QUANTUM MEMORY APPLICABILITY FOR QUANTUM REPEATERS

Quantum repeaters are a necessary component for the realization of long distance communication of quantum information. The implementation of such devices will enable the secure communication of information, paving the way for the quantum internet [22–24]. The need for quantum repeaters stems from the limitations on information transfer distance in optical fibers. Photons are superb 'flying' qubits that can be used for long distance communication of quantum states owing to their long coherence times. Yet modern optical fibers can only be used to transmit photons for distances of $\approx 20 - 100$ km due to attenuation. One solution to this problem is to transmit the quantum information via satellites, removing the issue of losses due to fiber attenuation. Such systems have been demonstrated, enabling quantum teleportation over distances of up to 1400 km, yet issues such as sensitivity to atmospheric turbulence and the requirement for direct line of sight with the satellite remain [25]. Another alternative is to use quantum repeaters, which seek to get around this limitation by splitting the full communication distance into several shorter channels

connected by quantum repeater nodes, by entangling all of the nodes, entanglement swapping can then be used to transmit information between the two end nodes over distances greater than those possible by direct photon transmission [22–24, 26]. This simplified approach requires that entanglement generation occurs on every repeater node simultaneously, which significantly limits the chance of success. By using a quantum memory qubit which possess a long coherence time T_2, the entangled states can be stored for time T_2 allowing multiple attempts at generating entanglement. Therefore, by using time multiplexing, the chance of successful entanglement swapping can be increased [24, 27].

The storage time of the memory required to achieve the transfer of information over a useful distance varies significantly depending on the quantum repeater protocol. In particular, the required communication time can be reduced by introducing more complexity to the system, such as multiplexing [28, 29]. Therefore the memory time needed for a $\approx$ 1000 km channel can vary from 1000 s down to 1 ms [22, 23, 30]. Fundamentally, the maximum distance achievable for a given memory time is tied to the speed of light, therefore memories of 1 ms are sufficient to beat direct photon transmission [23] while 5 ms is sufficient to reach distances of 1000 km, a commonly used benchmark in the literature [22]. Therefore, using this approach, we estimate that a quantum memory based on nuclear spins with $T_{\mathrm{M}} \approx 100$ ms would be able to store a given state for long enough to send a message over a distance greater than half the Earth's circumference, meaning it would be sufficient for world wide communication.

However, photon transmission time is not the only limitation of a quantum repeater [30, 31]. Other limiting factors include: light-matter entanglement generation [32–34], state transfer to the nuclei [35, 36], entanglement swapping [37], and state measurement [38]. While all these building blocks have been demonstrated for QDs, they would all consume time, reducing the amount of time for generating entanglement. Out of the factors listed above, the generation of entanglement is the main limitation on communication distance. In the above discussion we assumed a unity fidelity of entanglement generation. In reality this is not the case and the photon transmission process needs to be repeated multiple times until the entanglement is established, which significantly limits the achievable distances for quantum repeaters [39, 40]. For QDs, entanglement generation rates as high as 7.3 kHz have been demonstrated [37]. We can therefore estimate, using the equations and parameters reported in Ref. [40], that with the current memory storage time of $T_{\mathrm{M}} \approx 100$ ms it should be possible to achieve quantum repeater communication distances of $\approx$ 13.4 km. Assuming a link efficiency (defined as the ratio of memory lifetime over entanglement generation time [39]) of 0.34, as used in [40] leads to a more favorable estimate of $\approx$ 39.4 km, making nuclear spin based

memories competitive with the atomic ensemble systems used in Ref. [40], as well as matching communication distances achievable with direct fiber transmission of photons.

Although current nuclear spin memory times do not yet match those achievable in certain solid state or atomic systems [41–43], the III-V semiconductor quantum dots benefit from superior optical properties such as efficient generation of single [44–47] and entangled photons [48, 49], which are also required for quantum communication. QDs also benefit from the well established semiconductor fabrication techniques, offering a path for scalability. The storage times demonstrated here for nuclear spins, combined with the high optical entanglement generation rates mean that QDs should be able to compete with other existing approaches to quantum repeaters [37, 40]. Finally, by using multiplexing or alternatively, by extending the coherence time to $T_2 = 1$ s as discussed in the main text, it should be possible to extend the possible communication distance to $500 - 1000$ km, sufficient to outperform direct photon transmission and enable quantum repeater channels that, for example, connect major cities [28, 30].

Supplementary Note 5. AVERAGE HAMILTONIAN THEORY APPLIED TO DESIGN OF CHASE DYNAMICAL DECOUPLING SEQUENCES

A. Nuclear spin interactions

The Zeeman term accounts for the coupling of the QD nuclear spins $\mathbf{I}_k$ to the static magnetic field B_z directed along the z axis. In the laboratory frame it can be written as

$$\mathcal{H}_{\mathrm{Z,N}} = -\sum_{k=1}^{N} \hbar\gamma_k B_\mathrm{z} \hat{I}_{\mathrm{z},k}, \tag{S1}$$

where the summation goes over all individual nuclei $1 \leq k \leq N$, $\hbar = h/(2\pi)$ is the reduced Planck's constant, γ_k is the gyromagnetic ratio of the k-th nuclear spin and $\hat{\mathbf{I}}_k$ is a vector of spin operators with Cartesian components $(\hat{I}_{\mathrm{x},k}, \hat{I}_{\mathrm{y},k}, \hat{I}_{\mathrm{z},k})$. The result of the Zeeman term alone is a spectrum of equidistant single-spin eigenenergies $-I_\mathrm{z}\hbar\gamma_k B_\mathrm{z}$. These $2I + 1$ states are also the eigenstates of the $\hat{I}_\mathrm{z}$ spin projection operator with eigenvalues I_z satisfying $-I \leq I_\mathrm{z} \leq +I$.

The interaction of the nuclear electric quadrupolar moment with the electric field gradients is described by the term (Ch. 10 in Ref. [50]):

$$\mathcal{H}_{\mathrm{Q,N}} = \sum_{k=1}^{N} \frac{q_k}{6}[3\hat{I}^2_{\mathrm{z}',k} - I^2_k + \eta_k(\hat{I}^2_{\mathrm{x}',k} - \hat{I}^2_{\mathrm{y}',k})], \tag{S2}$$

where q_k and η_k describe the magnitude and asymmetry of the electric field gradient tensor, whose principal axes are $x'y'z'$. The strain is inhomogeneous within the QD volume, so that q_k and η_k

vary between the individual nuclei. The axes $x'y'z'$ are different for each nucleus and generally do not coincide with crystallographic axes or magnetic field direction. For the as-grown (unstrained) GaAs/AlGaAs QDs the typical quadrupolar shift is around $|q_k|/h \approx 24$ kHz for ^{75}As [1, 11]. The full width at half maximum of the $|q_k|/h$ distribution is ≈ 14 kHz. For a small fraction of arsenic nuclei, adjacent to aluminium atoms, the shifts are as large as $|q_k|/h \approx 200$ kHz [2]. In the strained sample structure, q_k are dominated by the extrinsic uniaxial stress, with the typical values $|q_k|/h \approx 250$ kHz for ^{75}As in the present work. All experiments are conducted under sufficiently strong magnetic fields, where $|\hbar\gamma_k B_{\mathrm{z}}| \gg |q_k|$ and quadrupolar effects can be treated perturbatively. In this perturbative regime, the main effect of the quadrupolar shifts is the anharmonicity of the nuclear spin eigenenergies and the resulting quadrupolar NMR multiplet of $2I$ magnetic-dipole transitions, split by $\nu_{\mathrm{Q}} \approx q_k/h$. The $I_{\mathrm{z}} = \pm 1/2$ projection states of a half-integer nuclear spin are influenced by quadrupolar effects only in the second order. These second order shifts scale as $\propto \nu_{\mathrm{Q}}^2/\nu_{\mathrm{N}}$, where $\nu_{\mathrm{N}} = \gamma B_{\mathrm{z}}/(2\pi)$ is the nuclear spin Larmor frequency.

Since we apply dynamical decoupling only to the two-level subspaces of the spin 3/2 four-level Hilbert space, we can use the standard rotating frame representation. For the $I_{\mathrm{z}} = \pm 1/2$ subspace the reference frequency of the frame is set to the Larmor frequency ν_{N}, while for the satellite subspaces the reference frequency is set to $\nu_{\mathrm{N}} \pm \nu_{\mathrm{Q}}$ to match the NMR frequency of the corresponding satellite transition. The rotating frame transformation in the following effective Zeeman Hamiltonian term

$$\mathcal{H}'_{\mathrm{Z,N}} = -\sum_{k=1}^{N} h\Delta\nu_k \hat{I}_{\mathrm{z},k}, \tag{S3}$$

where $\Delta\nu_k$ is the resonance offset of the kth nucleus. In the case of the $I_{\mathrm{z}} = \pm 1/2$ spin subspace, $\Delta\nu_k$ represents the inhomogeneous second order quadrupolar shifts, which are within $\lesssim 1$ kHz. For the $I_{\mathrm{z}} = (-3/2, -1/2)$ satellite transition subspace the set of $\Delta\nu_k$ describes the distribution of the first order quadrupolar shifts which arise from inhomogeneous strain within the QD volume and range from tens to hundreds of kHz for the individual ^{75}As nuclei.

Direct interaction between the nuclei is described by the dipole-dipole Hamiltonian:

$$\begin{aligned} \mathcal{H}_{\mathrm{DD}} &= \sum_{1 \le j < k \le N} b_{j,k} \left(3\hat{I}_{\mathrm{z},j}\hat{I}_{\mathrm{z},k} - \hat{\mathbf{I}}_j \cdot \hat{\mathbf{I}}_k \right), \\ b_{j,k} &= \frac{\mu_0 \hbar^2}{4\pi} \frac{\gamma_j \gamma_k}{2} \frac{1 - 3\cos^2\theta_{j,k}}{r_{j,k}^3} \end{aligned} \tag{S4}$$

Here, $\mu_0 = 4\pi \times 10^{-7}$ N A^{-2} is the magnetic constant and $r_{j,k}$ denotes the length of the vector, which forms an angle θ with the z axis and connects the two spins j and k. The Hamiltonian

of Supplementary Eq. S4 has been truncated to eliminate all spin non-conserving terms – this is justified for static magnetic field exceeding $\gtrsim 1$ mT. The spin-spin interaction constants can be written in frequency units $\nu_{jk} = b_{j,k}/h$. The typical magnitude of the interaction constants for the nearby nuclei in GaAs is $\max|\nu_{jk}| \approx 100$ Hz. Consequently, the typical timescales of the decoherence driven by the many-body dipole-dipole interactions are on the order of $T_2 \approx 1$ ms. The dipole-dipole interaction does not change its form under rotating frame transformation.

B. Average Hamiltonian theory

The basics of average Hamiltonian theory can be found in relevant textbooks [51]. Here we briefly outline the key points. The effect of the Rf pulses can be viewed as transformation of the many-body nuclear spin Hamiltonian. In the limit of short and strong Rf pulses, we can treat the Hamiltonian in a toggling reference frame as a piece-wise function of time $\tilde{\mathcal{H}}(t)$, which remains constant in between the Rf pulses. After the first pulse, the Hamiltonian can be written as:

$$\mathcal{H}_1 = P_1^{-1}\mathcal{H}_0 P_1, \tag{S5}$$

where $\mathcal{H}_0 = \mathcal{H}'_{\mathrm{Z,N}} + \mathcal{H}_{\mathrm{DD}}$ is the initial Hamiltonian in the rotating frame and P_1 is the unitary transformation operator. After the second and third Rf pulses, the Hamiltonian is:

$$\mathcal{H}_2 = (P_2P_1)^{-1}\mathcal{H}_0 P_2 P_1, \tag{S6}$$

$$\mathcal{H}_3 = (P_3P_2P_1)^{-1}\mathcal{H}_0 P_3 P_2 P_1, \tag{S7}$$

and so on for the subsequent pulses. The evolution of the spin ensemble can in principle be calculated using the Hamiltonians, such as in equations S5, S7, to derive the unitary propagators in each time segment. However, for long pulse sequences this quickly becomes impractical. Instead, we want to find the propagator over the entire pulse sequence cycle T_{Cycle}

$$U(T_{\mathrm{Cycle}}) = \exp\left(-iT_{\mathrm{Cycle}}\mathcal{H}_{\mathrm{eff}}\right), \tag{S8}$$

where $\mathcal{H}_{\mathrm{eff}}$ is some effective Hamiltonian describing spin interactions in the toggling frame over the pulse sequence cycle. Such an effective Hamiltonian can indeed be found in the form of a Magnus expansion

$$\mathcal{H}_{\mathrm{eff}} = \mathcal{H}^{(0)} + \mathcal{H}^{(1)} + \mathcal{H}^{(2)}..., \tag{S9}$$

where the first terms of the expansion are [51]:

$$\mathcal{H}^{(0)} = \frac{1}{T_{\mathrm{Cycle}}} \int_0^{T_{\mathrm{Cycle}}} \tilde{\mathcal{H}}(t)\mathrm{d}t \,, \tag{S10}$$

$$\mathcal{H}^{(1)} = \frac{-i}{2T_{\mathrm{Cycle}}} \int_0^{T_{\mathrm{Cycle}}} \mathrm{d}t_2 \int_0^{t_2} \mathrm{d}t_1 \left[\tilde{\mathcal{H}}(t_2), \tilde{\mathcal{H}}(t_1)\right] \,, \tag{S11}$$

$$\mathcal{H}^{(2)} = \frac{-1}{6T_{\mathrm{Cycle}}} \int_0^{T_{\mathrm{Cycle}}} \mathrm{d}t_3 \int_0^{t_3} \mathrm{d}t_2 \int_0^{t_2} \mathrm{d}t_1 \Big(\left[\tilde{\mathcal{H}}(t_1), \left[\tilde{\mathcal{H}}(t_2), \tilde{\mathcal{H}}(t_3)\right]\right] + \left[\tilde{\mathcal{H}}(t_3), \left[\tilde{\mathcal{H}}(t_2), \tilde{\mathcal{H}}(t_1)\right]\right] \Big) \,. \tag{S12}$$

The $\mathcal{H}^{(0)}$ term is the average Hamiltonian over the pulse sequence cycle. In all CHASE sequences it is eliminated $\mathcal{H}^{(0)} = 0$ in the limit of short Rf control pulses $T_{\mathrm{RF}} \to 0$. For the term $\mathcal{H}^{(n)}$, the integration volume scales as $\propto T_{\mathrm{Cycle}}^{n+1}$. Thus, as long as $\mathcal{H}^{(0)} = 0$, one can ensure the convergence of the effective Hamiltonian to zero $\mathcal{H}_{\mathrm{eff}} \to 0$ in the limit of a short cycle $T_{\mathrm{Cycle}} \to 0$ (i.e. in the limit of fast dynamical decoupling). This is the main goal of the time-suspension dynamical decoupling. As a general rule, one seeks to eliminate the lowest order terms in the expansion, in order to achieve faster convergence of the effective Hamiltonian.

C. Average Hamiltonians of the CHASE cycles

We have calculated average Hamiltonian terms of the CHASE sequences up to the second order in Magnus expansion, inclusive. We take into account the finite (nonzero duration, $T_{\mathrm{Rf}} > 0$) Rf pulses, which represent the time segments within the sequence cycle where the toggling frame Hamiltonian depends explicitly on time. For simplicity, we only treat Rf pulses with rectangular envelope function. Although this is different from the raised cosine pulses used in experiment, the rectangular pulses are sufficient to derive the main effect of the finite control pulses on the average Hamiltonian terms.

We start with the CHASE-10 sequence cycle. The leading term appears in the average Hamiltonian $\mathcal{H}^{(0)}$ under finite pulses $T_{\mathrm{Rf}} > 0$. This Hamiltonian term has the form of a dipole-dipole interaction (Equation S4) but with an effective quantization axis along the y direction (i.e. $3\hat{I}_{\mathrm{z},i}\hat{I}_{\mathrm{z},j}$ is replaced with $3\hat{I}_{\mathrm{y},i}\hat{I}_{\mathrm{y},j}$):

$$\mathcal{H}^{(0)}_{\mathrm{CHASE-10}} = h\frac{T_{\mathrm{Rf}}}{T_{\mathrm{Cycle}}} \sum_{i<j} \nu_{ij}(3I_{\mathrm{y},i}I_{\mathrm{y},j} - \mathbf{I}_i \cdot \mathbf{I}_j). \tag{S13}$$

This interaction causes preferential spin locking of the nuclear spin states polarized along the y axis of the rotating frame. There are also non-zero first and second order terms in the Magnus expansion of the CHASE-10 effective Hamiltonian. The exact expressions can be derived, but

are bulky. These higher order terms can be neglected, since they appear only for finite Rf pulses $T_{\rm Rf} > 0$ and are dominated by the zero order term $\mathcal{H}^{(0)}$ in the practically useful limit of a short cycle time $T_{\rm Cycle}$.

The expression for the dominant zero order term of CHASE-20 is twice that of CHASE-10 (Eq. S13). However, since $T_{\rm Cycle}$ appears in denominator of Eq. S13 and is twice longer for CHASE-20, the resulting zero order average Hamiltonian is the same as for CHASE-10. CHASE-20 is a symmetrised version of CHASE-10, i.e. it consists of a CHASE-10 subcycle and second CHASE-10 subcycle where all pulses are applied in reverse order. Symmetrisation leads to cancellation of the first order Magnus term, as well as all other terms with odd orders.

We now consider CHASE-40, the new sequence implemented in this work. The zero order (average) Hamiltonian term appears only under finite pulses $T_{\rm Rf} > 0$ and has the following form

$$\mathcal{H}^{(0)}_{\rm CHASE-40} = -2h\frac{T_{\rm Rf}}{T_{\rm Cycle}}\sum_{i<j}\nu_{ij}(3I_{{\rm z},i}I_{{\rm z},j} - \mathbf{I}_i\cdot\mathbf{I}_j) \tag{S14}$$

Compared to Eq. S13, there is an additional factor of 2, but $T_{\rm Cycle}$ in the denominator is 4 times larger. Thus the $\mathcal{H}^{(0)}_{\rm CHASE-40}$ term is twice smaller in magnitude compared to $\mathcal{H}^{(0)}_{\rm CHASE-10}$. Moreover, the symmetry axis of $\mathcal{H}^{(0)}_{\rm CHASE-40}$ coincides with the external magnetic field (z axis). Thus, there is no preferential direction in the equatorial plane of the rotating frame, explaining the absence of spin locking.

CHASE-40 is not symmetric, so the first order Magnus term is not canceled. However, for all physically relevant parameters the first order term is found to be dominated, either by the zero order term (at short $T_{\rm Cycle}$) or by the second order term discussed below (for long $T_{\rm Cycle}$). The small first order term is not a major issue since it can be easily eliminated with an 80-pulse symmetrised supercycle [51, 52].

The second order term of CHASE-40 is present even for ideal Rf pulses $T_{\rm Rf} \to 0$. Thus we only consider this limit of delta-pulses and ignore all the corrections arising from finite pulses $T_{\rm Rf} > 0$. Even then, the expression for the second order term of CHASE-40 is rather bulky. There are different types of contributions. In particular there are the so called cross terms which appear only in presence of both the resonance offset of the individual spins ($\Delta\nu_k \neq 0$) and the dipole-dipole interactions between the spins ($\nu_{jk} \neq 0$). These cross terms play an important role for the $I_{\rm z} = (-3/2, -1/2)$ spin subspace subject to a considerable first order quadrupolar broadening, leading to $\Delta\nu_k$ on the order of tens of kHz. For the homogeneous $I_{\rm z} = \pm 1/2$ subspace, affected only by the second order quadrupolar shifts, the resonance offsets $\Delta\nu_k$, and hence the cross term in $\mathcal{H}^{(2)}_{\rm CHASE-40}$, can be made arbitrarily small, for example by increasing the static magnetic field,

which increases the nuclear Larmor frequency $\nu_{\rm N}$. But even in the absence of any resonance offsets $\Delta\nu_k = 0$, there remains a second order Magnus term of the following form

$$\mathcal{H}^{(2)}_{\rm CHASE-40} = h\frac{T^2_{\rm Cycle}}{110592}\sum_{i<j}\Big[\big(3\sum_{k\neq i,j}(\nu_{ik}+\nu_{jk})(\nu_{ik}\nu_{jk}+\nu_{ij}\nu_{ik}+\nu_{ij}\nu_{jk})\big)+ \\ +8\nu_{ij}(\Delta\nu_i^2+\Delta\nu_i\Delta\nu_j+\Delta\nu_j^2)\Big](3I_{{\rm z},i}I_{{\rm z},j}-\mathbf{I}_i\cdot\mathbf{I}_j) \qquad \text{(S15)}$$

This term has a form of an effective dipolar coupling between spins i and j, summed over all unique pairs $i < j$. Such interaction can arise from the direct dipole-dipole interaction of spins i and j in presence of a resonance offset for at least one of the spins from the pair (the $8\nu_{ij}(\Delta\nu_i^2+\Delta\nu_i\Delta\nu_j+\Delta\nu_j^2)$ term). However, the interaction remains even if all $\Delta\nu_k = 0$, but requires dipolar coupling to some third spin k. Such interaction can be interpreted as a three particle effect, where the effective interaction of spins i and j is mediated by all other spins $k \neq i, j$. There are some further second order terms that appear only under non-zero resonance offsets, but these are small and are omitted in Eq. S15.

The $\mathcal{H}^{(2)}_{\rm CHASE-40}$ term of Eq. S15 dominates the residual toggling-frame Hamiltonian of CHASE-40 in the limit of large $T_{\rm Cycle}$. It is responsible for the quadratic growth of the decoherence rate $T_{\rm M}^{-1}$ with increasing $T_{\rm Cycle}$ at large $T_{\rm Cycle} \gtrsim 2$ ms, as observed in Fig. 3e of the main text for the measurement on the CT subspace $I_{\rm z} = \pm 1/2$. The zero order term $\mathcal{H}^{(0)}_{\rm CHASE-40}$ (Eq. S14) is negligible at large $T_{\rm Cycle}$ due to the small ratio $T_{\rm Rf}/T_{\rm Cycle}$, but becomes dominant in the limit of fast dynamical decoupling (when most of the sequence cycle is taken up by the control pulses, corresponding to $T_{\rm Rf}/T_{\rm Cycle} \approx 1/40$). The zero order term $\mathcal{H}^{(0)}$ is responsible for the growth of the decoherence rate $T_{\rm M}^{-1}$ with reducing $T_{\rm Cycle}$ at small $T_{\rm Cycle} \gtrsim 2$ ms, as observed in Fig. 3e of the main text for the measurement on the CT subspace $I_{\rm z} = \pm 1/2$. The slowest decoherence (the longest spin memory time $T_{\rm M}$) is achieved when the contributions of the zero and second order terms are comparable. This observation allows us to estimate the maximum $T_{\rm M}$ that would be achieved if the effect of finite pulses was eliminated [52, 53]. Assuming the second order term would be unaffected, we can extrapolate the measured power-law dependence of $T_{\rm M}$ on $T_{\rm Cycle}$ at large $T_{\rm Cycle} \gtrsim 2$ ms into the range of small $T_{\rm Cycle}$. This yields an expected $T_{\rm M} \approx 1$ s for the shortest possible $T_{\rm Cycle} = 0.8$ ms of a CHASE-40 cycle at $T_{\rm RF} = 20$ μs used in this work. This prediction shows that another order of mangitude improvement in the quantum memory storage time might be within reach with the existing material parameters (such as strain) and using only pulse sequence design techniques [52, 53].

Even once the effective Hamiltonian (Eq. S9) is known, finding the spin dynamics is still a

difficult problem. Formally, the relaxation function of the transverse nuclear spin polarization can be written as an infinite power series over time, where the coefficients are the moments of the NMR spectral lineshape (Ch. 6 in [54]). In practice, only the second (M_2) and the fourth (M_4) moments can be calculated [55], making the power series rather inaccurate. It is more practical to approximate the lineshape with a function, such as Gaussian, and use the second moment M_2 as a linewidth parameter. The approximate coherent memory time can then be found as

$$T_{\mathrm{M}} \approx \sqrt{2/M_2}. \tag{S16}$$

For nuclear spin polarization along the x axis of the rotating frame, the second moment can be caclualted as

$$M_2 = -\mathrm{Tr}\left\{[\mathcal{H}_{\mathrm{eff}}, \sum_{k=1}^{N} \hat{I}_{\mathrm{x},k}]^2\right\} / \mathrm{Tr}\left\{\left(\sum_{k=1}^{N} \hat{I}_{\mathrm{x},k}\right)^2\right\} \tag{S17}$$

The second moments can be calculated for an arbitrary orientation of the nuclear spin polarization by substituting the corresponding spin operators (e.g. $\hat{I}_{\mathrm{y},k}$) in Eq. S17.

For practical calculations, Eq. S17 is applied to a system of 3 nuclear spins, assuming the same dipolar coupling ν_{DD} between each spin pair, and the resonance offsets of $0, +\Delta\nu, -\Delta\nu$. The effective Hamiltonians are calculated for free induction decay (FID, no dynamical decoupling), Hahn echo (single π refocusing pulse), and CHASE-40, and are substituted into Eqns. S16, S17 to derive the spin memory times. The values for FID and Hahn echo are equated to the measured T_2 values (the difference between T_2 and T_{M} is negligible if there is at most one refocusing pulse). This allows the coupling parameters ν_{DD} and $\Delta\nu$ to be extracted and then used to calculate the spin memory time T_{M} for the CHASE-40 sequence. The resulting $T_{\mathrm{M}}^{\mathrm{CHASE-40}}$ is shown by the dashed lines as a function of T_{Cycle} in Fig. 3e of the main text and is discussed therein.

Supplementary Note 6. NUMERICAL MODELING OF NUCLEAR SPIN DECOHERENCE AND DYNAMICAL DECOUPLING

We perform exact numerical modeling on an ensemble of N spin-1/2 nuclei coupled through dipole-dipole interactions (Supplementary Eq. S4). The Zeeman effect of the strong static magnetic field is eliminated through rotating frame transformation. This leaves the Zeeman offset Hamiltonian (Supplementary Eq. S3), where $\Delta\nu_k$ is the resonance frequency shift of the k-th nucleus, which includes the inhomogeneous nuclear qaudrupolar effects and chemical shifts.

During the Rf pulses, the time-dependent Hamiltonian is added:

$$\mathcal{H}_{\mathrm{Rf,N}} = -h \sum_k \nu_1(t) \left(\cos(\phi) \hat{I}_{\mathrm{x},k} - \sin(\phi) \hat{I}_{\mathrm{y},k} \right), \quad \text{(S18)}$$

where the summation goes over all nuclei, $\nu_1(t)$ is the slowly-varying Rf pulse envelope, and ϕ describes the phase of the Rf carrier and the corresponding orientation of the transverse magnetic field in the equatorial plane of the rotating frame. The envelope function has a raised cosine profile $\nu_1(t) \propto (1 - \cos(2\pi(t - t_0)/T_{\mathrm{Rf}}))/2$, where t_0 is the starting time of the Rf pulse burst.

The nuclei are arranged on a face-centered cubic (FCC) lattice. The $\{x, y, z\}$ lattice coordinates, ordered by their proximity to the $\{x, y, z\} = \{0, 0, 0\}$ point, are given below in units of nm:

$$\begin{aligned}
&\{0., 0., 0.\}; \{0.282393, 0.282393, 0.\}; \{0.282393, 0., 0.282393\}; \{0.282393, -0.282393, 0.\};\\
&\{0.282393, 0., -0.282393\}; \{0., 0.282393, 0.282393\}; \{-0.282393, 0.282393, 0.\};\\
&\{0., 0.282393, -0.282393\}; \{-0.282393, 0., 0.282393\}; \{0., -0.282393, 0.282393\};\\
&\{-0.282393, -0.282393, 0.\}; \{-0.282393, 0., -0.282393\}; \{0., -0.282393, -0.282393\};\\
&\{0.564786, 0., 0.\}; \{0., 0.564786, 0.\}; \{0., 0., 0.564786\};\\
&\{0., 0., -0.564786\}; \{0., -0.564786, 0.\}; \{-0.564786, 0., 0.\};\\
&\{0.282393, 0.282393, 0.564786\}; \{0.282393, 0.564786, 0.282393\}; \{0.564786, 0.282393, 0.282393\}.
\end{aligned} \quad \text{(S19)}$$

Most of the numerical modeling is carried out for a system of $N = 12$ ^{75}As nuclear spins, in which case the lattice is constructed by taking the first 12 points from Supplementary Eq. S19.

The unitary evolution of the system is simulated through numerical propagation of the Schrödinger equation from an initial wave function state ψ_{Init}. The computation is carried out using the software package Wolfram Mathematica 13.2 and the Python QuTiP 4.7.3 package [56]. The procedure depends on the type of the initial state. When analysing the dynamical decoupling experiments (Figs. 2, 3, and 4 of the main text), the initial states are chosen to mimic the states with macroscopic nuclear polarization used in the experiments. We chose ψ_{Init} as eigenstates of the time-independent Hamiltonian, which is a sum of Supplementary Eq. S4 and Supplementary Eq. S3. We typically use 16 different initial eigenstates, spanning the whole range of the total spin z projections from $-N/2$ to $+N/2$. Once the spin evolution is calculated for a particular Rf pulse sequence, we use the final wavefunction ψ_{Fin} to calculate the final nuclear spin polarization $I_{\mathrm{z,Fin}} = \langle \psi_{\mathrm{Fin}} | \sum_{k=1}^{N} \hat{I}_{\mathrm{z},k} | \psi_{\mathrm{Fin}} \rangle$. Each final polarization value is normalized by the sign of the initial polarization $I_{\mathrm{z,Init}}$, and these normalized values are then averaged over all the initial

states $\psi_{\rm Init}$. Such averaging over multiple initial coherent states corresponds to a mixed initial state and helps to eliminate the oscillations that arise due to the small number of spins N in the model. This averaging also mimics the experimental conditions, where the initial optically-induced nuclear polarization is subject to uncertainty due to fluctuations and drifts of various experimental parameters (e.g. optical pump power). Following the averaging over the initial states, the final polarization values are normalized by the average initial polarization. This way $I_{\rm z,Fin} = 1$ corresponds to vanishingly small decay of the initial nuclear spin polarization (see e.g. Fig. 2d of the main text). The exact same set of initial wavefunction states is used when sweeping the parameters of the Rf pulse sequence, which ensures repeatability of the spin dynamics.

When performing numerical modeling of spin wave dynamical decoupling (Fig 5 of the main text), the initial states are chosen as pure states. The state with full longitudinal polarization, which is a pure ground state $|G\rangle = |\uparrow_1 \otimes \uparrow_2 \otimes ... \otimes \uparrow_N\rangle$, is constructed as a Kronecker product of single-particle states, where each (ith) nuclear spin is in a single-particle basis state $|\uparrow_i\rangle$ with projection $I_{\rm z} = 1/2$ onto the static magnetic field. A single-quantum spin wave excitation of the ground state is the generalized W state, which is a superposition of all possible products where one of the spins is in a $|\downarrow_i\rangle$ single-particle basis state with $I_{\rm z} = -1/2$ projection, with all other spins in a $|\uparrow\rangle$ single-particle basis state [57]: $|W\rangle = N^{-1/2}\sum_{i=1}^{N}|\uparrow_1 \otimes \uparrow_2 \otimes ... \otimes \downarrow_i \otimes ... \otimes \uparrow_N\rangle$. The spin wave superposition initial state (open symbols in Fig. 5 of the main text) is then constructed as a superposition of the ground state and the W-state: $2^{-1/2}(|G\rangle + e^{i\alpha}|W\rangle)$. Such state can be created through coherent coupling of the central electron spin to the nuclear spin ensemble [36], imprinting the phase of the electron spin into the phase α of the spin wave. The results shown in Fig. 5 of the main text are for $\alpha = 0$. Numerical modeling with $\alpha = \pi/2$ gives very similar results. For comparison, we perform numerical modeling with a multiple-quantum pure initial state (solid symbols in Fig. 5 of the main text), which is constructed as a Kronecker product of superpositions of $I_{\rm z} = \pm 1/2$ single-particle basis states: $|X\rangle = 2^{-N/2}|(\uparrow_1 + \downarrow_1) \otimes (\uparrow_2 + \downarrow_2) \otimes ... \otimes (\uparrow_N + \downarrow_N)\rangle$. This state corresponds to full transverse polarization (macroscopic magnetization) of a nuclear spin ensemble and can be created by optically pumping [3] the nuclear spin ensemble into the ground state $|G\rangle$ and then applying a non-selective $\pi/2$ Rf rotation pulse. For numerical modeling with pure initial states we characterize decoherence by calculating the overlap probability $|\langle\psi_{\rm Init}|\psi_{\rm Fin}\rangle|^2$ of the initial and final states.

When modeling the nuclear spin dynamics of the homogeneous $I_{\rm z} = \pm 1/2$ subspace we set all resonance offsets to be zero $\Delta\nu_k = 0$. For the $I_{\rm z} = (-3/2, -1/2)$ satellite transition subspace we model the resonance offset of each nuclear spin k as $\Delta\nu_k = \Delta\nu_0(k-1) + {\rm N}(0, 0.2\Delta\nu_0/(2\sqrt{2\ln 2}))$,

where $\mathrm{N}(\mu, \sigma)$ stands for a normal distribution with the mean μ and standard deviation σ. This model ensures that the resonance frequency of each nucleus is detuned from the nearest frequency of another nucleus by $\Delta\nu_0$, on average. Each resonance shift is randomly varied (by a $\approx 20\%$ fraction of $\Delta\nu_0$) to avoid periodic recurrences and oscillations. Once the set of randomized $\Delta\nu_k$ is generated, it is fixed and used throughout the different modeling runs. We have used three sets with $\Delta\nu_0 = 0.8$ kHz, $\Delta\nu_0 = 1.2$ kHz, and $\Delta\nu_0 = 1.6$ kHz. The calculated final nuclear spin polarizations $I_{\mathrm{z,Fin}}$ are averaged over these three sets, once again to combat the oscillations that arise from the small number of spins N in the model.

The numerical modeling with mixed initial states closely follows the experiments: the total free evolution time T_{FreeEvol} and the number of cycles n_{Cycles} of the dynamical decoupling sequence are varied, to calculate the final nuclear spin polarization $I_{\mathrm{z,Fin}}$, averaged over initial states and resonance offsets as described above. The dependencies on T_{FreeEvol} yield decay curves that are similar to the experimental curves shown in Fig. 2c of the main text. Alternatively, the same data can be plotted as a function of the total evolution time T_{EvolTot} and the decoupling sequence period T_{Cycle}, such as shown in Figs. 3c, d of the main text. The numerically modeled values of the final nuclear polarization $I_{\mathrm{z,Fin}}$ shown in Figs. 3c, d of the main text are normalized by the initial nuclear polarization $I_{\mathrm{z,Init}}$ averaged over all the initial states. This way, the normalized values are limited to the $[-1, +1]$ range, with $+1$ corresponding to no decay of the nuclear spin polarization (i.e. complete preservation through dynamical decoupling). The values close to 0 can be interpreted as complete decoherence, whereas the negative values of the normalized nuclear spin echo amplitude are a sign of recurrences in the dynamics of a small nuclear spin ensemble. The corresponding experimental data, shown in Figs. 3a, b of the main text, is also normalized, but using the amplitude of the simple Hahn echo decoupling measured at short T_{FreeEvol}, which approximates the initial nuclear spin polarization with good accuracy.

Supplementary Fig. 8 shows nuclear spin coherence times T_2 derived from numerical modeling on spin ensembles with a variable number N of dipolar coupled nuclei. In this study we consider a homogeneous spin ensemble, where all resonance offsets are set to zero ($\Delta\nu_k = 0$). Under simple Hahn echo decoupling (squares), T_2 is seen to decrease monotonically with increasing N. For N increasing from 4 to 14, T_2 reduces monotonically by a factor of ≈ 1.64. This accelerated decoherence can be explained by an increasing number of pairwise spin interactions in an ensemble with larger N. The coherence times T_2 under CHASE-40 dynamical decoupling with $n_{\mathrm{Cycles}} =$ 4 cycles are shown by the circles in Supplementary Fig. 8 . Unlike with Hahn echo, there is no clear trend in $T_2(N)$ dependence for CHASE-40. The T_2 time under CHASE-40 varies in a

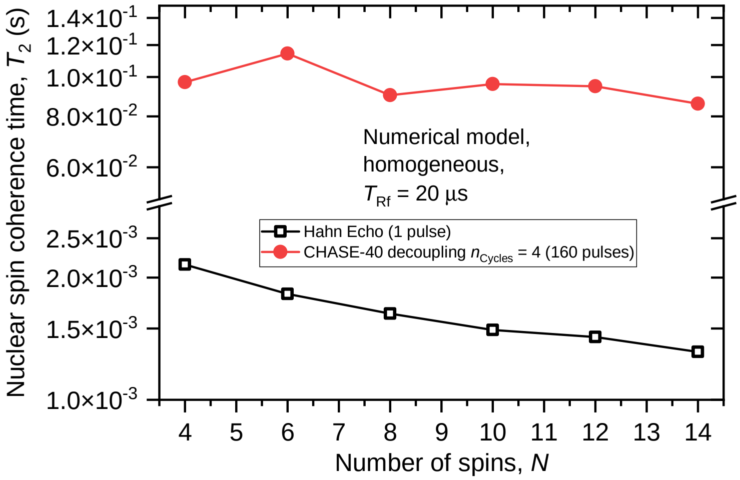

Supplementary Figure 8. **Dependence of numerical modeling results on number of spins.** Decoherence under dynamical decoupling derived from first-principles numerical modeling of spin dynamics of a homogeneous ensemble with a variable number N of nuclear spins. Results are shown for Hahn echo (squares) and $n_{\text{Cycles}} = 4$ cycles of CHASE-40 (circles).

narrower range, changing by a factor of ≈ 1.33 for $4 \leq N \leq 14$. The limited range of available N (restricted by the exponential growth in computational resources required for exact numerical modeling) precludes a more detailed study. Based on the available data, we put forward the following explanation. The decoherence under Hahn echo is governed by the direct (pairwise) dipole-dipole interaction of the nuclear spins. The larger the N, the larger is the number of pairs and the shorter is the coherence time. By contrast, CHASE-40 eliminates the direct pairwise interactions, so that the decoherence is dominated by the residual effective three-spin interaction, and possibly interactions involving even higher particle numbers. The interplay of these weak residual many-body couplings is likely to be behind the irregular dependence of T_2 on N seen in Supplementary Fig. 8. The same reasons can be behind the discrepancy in the CHASE coherence times, which are found to be considerably longer in numerical modeling than in experimental NMR. It is possible that numerical modeling on much larger ensembles with $N \gg 12$ would accurately capture the complexity of the three-spin interactions and yield a reduction of T_2 towards the measured values at large N. However, exact modeling of systems with $N \gg 12$ is currently beyond reach with the available standard workstation computers or general-purpose high-performance clusters.

Approximate methods, such as cluster expansion techniques [58, 59], could be an alternative approach to model large spin ensembles. However, these approximate expansion methods typically

work by keeping the leading orders of interactions, while truncating the small residual interactions. The dynamical decoupling works essentially on the opposite principle of averaging out the leading interaction terms to zero, so that the resulting spin dynamics are governed by the weak residual interactions. The better the dynamical decoupling sequence, the more complex is the nature of the residual interaction terms and the larger is the number of such residual terms. Thus, achieving good convergence in approximate models might be challenging when dealing with efficient dynamical decoupling protocols. Further work would be needed to bridge the gap between exact modeling of small spin ensembles ($N \approx 10$) and experiments on real-sized nuclear spin ensembles ($N \approx 10^4$).

Supplementary Note 7. RAW DATA

Raw data is provided in text files using a tab-separated format (`.tsv`) and duplicated in spreadsheet format (`.xls`). The first line in each text file contains the headers.

The main experimental dataset used in Figs. 3a,b of the main text is tabulated in files `Fig3a.tsv` and `Fig3b.tsv`, respectively. The data shown in Fig. 2c of the main text is plotted from the same dataset as Fig. 3a. Each line in a text file corresponds to a single measurement of the nuclear spin echo decay under dynamical decoupling. The settings of the decoupling pulse sequence are given in two complementary formats. The sequence can be fully described by the sequence cycle type, the number of cycles n_{Cycles} and the free evolution time T_{FreeEvol}, as done in Fig. 2c of the main text. Alternatively, the experiment can be fully defined by the total evolution time T_{EvolTot} and the cycle time T_{Cycle} of the CHASE-40 sequence (or an equivalent value for shorter cycles), as done in Figs. 3a,b of the main text. The measured value of the nuclear spin echo amplitude is given in μeV of the optically-detected variation in the electron hyperfine shift. The standard deviation over multiple measurements of the nuclear spin echo amplitude is also included for the Fig. 3a dataset.

The datasets from numerical modeling used in Figs. 3c,d of the main text are tabulated in files `Fig3c.tsv` and `Fig3d.tsv`, respectively. These computed datasets are formatted in the same way as the experimental datasets. The data shown in Fig. 2d of the main text is plotted from the same dataset as Fig. 3c.

The datasets used in other (one-dimensional) plots are also tabulated. The names of the corre-

sponding files match the names of the figures.

---

[1] Millington-Hotze, P., Manna, S., Covre da Silva, S. F., Rastelli, A. & Chekhovich, E. A. Nuclear spin diffusion in the central spin system of a GaAs/AlGaAs quantum dot. *Nat. Commun.* **14**, 2677 (2023).

[2] Zaporski, L. *et al.* Ideal refocusing of an optically active spin qubit under strong hyperfine interactions. *Nat. Nanotechnol.* **18**, 257–263 (2023).

[3] Millington-Hotze, P. *et al.* Approaching a fully-polarized state of nuclear spins in a solid. *Nat. Commun.* **15**, 985 (2024).

[4] Dyte, H. E. *et al.* Is wave function collapse necessary? explaining quantum nondemolition measurement of a spin qubit within linear evolution. *Phys. Rev. Lett.* **132**, 160804 (2024).

[5] Oshiyama, A. & Ohnishi, S. DX center: Crossover of deep and shallow states in Si-$Al_xGa_{1-x}As$. *Phys. Rev. B* **33**, 4320–4323 (1986).

[6] Mooney, P. M. Deep donor levels (DX centers) in III-V semiconductors. *J. Appl. Phys.* **67**, R1–R26 (1990).

[7] Zhai, L. *et al.* Low-noise GaAs quantum dots for quantum photonics. *Nat. Commun.* **11**, 4745 (2020).

[8] Heyn, C. *et al.* Highly uniform and strain-free GaAs quantum dots fabricated by filling of self-assembled nanoholes. *Appl. Phys. Lett.* **94**, 183113 (2009).

[9] Atkinson, P., Zallo, E. & Schmidt, O. G. Independent wavelength and density control of uniform GaAs/AlGaAs quantum dots grown by infilling self-assembled nanoholes. *J. Appl. Phys.* **112**, 4745 (2012).

[10] Huo, Y. H., Rastelli, A. & Schmidt, O. G. Ultra-small excitonic fine structure splitting in highly symmetric quantum dots on GaAs (001) substrate. *Appl. Phys. Lett.* **102**, 152105 (2013).

[11] Ulhaq, A. *et al.* Vanishing electron g factor and long-lived nuclear spin polarization in weakly strained nanohole-filled GaAs/AlGaAs quantum dots. *Phys. Rev. B* **93**, 165306 (2016).

[12] Gammon, D. *et al.* Electron and nuclear spin interactions in the optical spectra of single GaAs quantum dots. *Phys. Rev. Lett.* **86**, 5176–5179 (2001).

[13] Eble, B. *et al.* Dynamic nuclear polarization of a single charge-tunable InAs/GaAs quantum dot. *Phys. Rev. B* **74**, 081306 (2006).

[14] Skiba-Szymanska, J. *et al.* Overhauser effect in individual InP/$Ga_xIn_{1-x}P$ dots. *Phys. Rev. B* **77**, 165338 (2008).

[15] Ragunathan, G. *et al.* Direct measurement of hyperfine shifts and radio frequency manipulation of nuclear spins in individual CdTe/ZnTe quantum dots. *Phys. Rev. Lett.* **122**, 096801 (2019).

[16] Urbaszek, B. *et al.* Nuclear spin physics in quantum dots: An optical investigation. *Rev. Mod. Phys.* **85**, 79–133 (2013).

[17] Chekhovich, E., Hopkinson, M., Skolnick, M. & Tartakovskii, A. Suppression of nuclear spin bath

fluctuations in self-assembled quantum dots induced by inhomogeneous strain. *Nat. Commun.* **6**, 6348 (2015).

[18] Braun, P.-F. *et al.* Bistability of the nuclear polarization created through optical pumping in $In_{1-x}Ga_xAs$ quantum dots. *Phys. Rev. B* **74**, 245306 (2006).

[19] Waeber, A. M. *et al.* Pulse control protocols for preserving coherence in dipolar-coupled nuclear spin baths. *Nat. Commun.* **10**, 3157 (2019).

[20] Li, D. *et al.* Intrinsic origin of spin echoes in dipolar solids generated by strong π pulses. *Phys. Rev. B* **77**, 214306 (2008).

[21] Chekhovich, E. A. *et al.* Structural analysis of strained quantum dots using nuclear magnetic resonance. *Nat. Nanotechnol.* **7**, 646–650 (2012).

[22] Zhao, R. *et al.* Long-lived quantum memory. *Nat. Phys.* **5**, 100–104 (2009).

[23] Tittel, W. *et al.* Photon-echo quantum memory in solid state systems. *Laser Photonics Rev.* **4**, 244–267 (2010).

[24] Azuma, K. *et al.* Quantum repeaters: From quantum networks to the quantum internet. *Rev. Mod. Phys.* **95**, 045006 (2023).

[25] Ren, J.-G. *et al.* Ground-to-satellite quantum teleportation. *Nature* **549**, 70–73 (2017).

[26] Briegel, H.-J., Dür, W., Cirac, J. I. & Zoller, P. Quantum repeaters: The role of imperfect local operations in quantum communication. *Phys. Rev. Lett.* **81**, 5932–5935 (1998).

[27] Duan, L.-M., Lukin, M. D., Cirac, J. I. & Zoller, P. Long-distance quantum communication with atomic ensembles and linear optics. *Nature* **414**, 413–418 (2001).

[28] Collins, O. A., Jenkins, S. D., Kuzmich, A. & Kennedy, T. A. B. Multiplexed memory-insensitive quantum repeaters. *Phys. Rev. Lett.* **98**, 060502 (2007).

[29] Jiang, L., Taylor, J. M. & Lukin, M. D. Fast and robust approach to long-distance quantum communication with atomic ensembles. *Phys. Rev. A* **76**, 012301 (2007).

[30] Sharman, K., Kimiaee Asadi, F., Wein, S. C. & Simon, C. Quantum repeaters based on individual electron spins and nuclear-spin-ensemble memories in quantum dots. *Quantum* **5**, 570 (2021).

[31] Langenfeld, S., Thomas, P., Morin, O. & Rempe, G. Quantum repeater node demonstrating unconditionally secure key distribution. *Phys. Rev. Lett.* **126**, 230506 (2021).

[32] De Greve, K. *et al.* Quantum-dot spin–photon entanglement via frequency downconversion to telecom wavelength. *Nature* **491**, 421–425 (2012).

[33] Coste, N. *et al.* High-rate entanglement between a semiconductor spin and indistinguishable photons. *Nat. Photon.* **17**, 582–587 (2023).

[34] Laccotripes, P. *et al.* Spin-photon entanglement with direct photon emission in the telecom C-band. *Nat. Commun.* **15**, 9740 (2024).

[35] Gangloff, D. A. *et al.* Quantum interface of an electron and a nuclear ensemble. *Science* **364**, 62–66 (2019).

[36] Appel, M. H. *et al.* A many-body quantum register for a spin qubit. *Nat. Phys.* **21**, 368–373 (2025).

[37] Stockill, R. *et al.* Phase-tuned entangled state generation between distant spin qubits. *Phys. Rev. Lett.* **119**, 010503 (2017).

[38] Atatüre, M. *et al.* Quantum-dot spin-state preparation with near-unity fidelity. *Science* **312**, 551–553 (2006).

[39] Humphreys, P. C. *et al.* Deterministic delivery of remote entanglement on a quantum network. *Nature* **558**, 268–273 (2018).

[40] Yu, Y. *et al.* Entanglement of two quantum memories via fibres over dozens of kilometres. *Nature* **578**, 240–245 (2020).

[41] Wang, Y. *et al.* Single-qubit quantum memory exceeding ten-minute coherence time. *Nat. Photon.* **11**, 646–650 (2017).

[42] Wang, P. *et al.* Single ion qubit with estimated coherence time exceeding one hour. *Nat. Commun.* **12**, 233 (2021).

[43] Atatüre, M., Englund, D., Vamivakas, N., Lee, S.-Y. & Wrachtrup, J. Material platforms for spin-based photonic quantum technologies. *Nat. Rev. Mater.* **3**, 38–51 (2018).

[44] Neuwirth, J. *et al.* Quantum dot technology for quantum repeaters: from entangled photon generation toward the integration with quantum memories. *Mater. Quantum Technol.* **1**, 043001 (2021).

[45] Huber, D. *et al.* Highly indistinguishable and strongly entangled photons from symmetric GaAs quantum dots. *Nat. Commun.* **8**, 15506 (2017).

[46] Schweickert, L. *et al.* On-demand generation of background-free single photons from a solid-state source. *Appl. Phys. Lett.* **112**, 093106 (2018).

[47] Arakawa, Y. & Holmes, M. J. Progress in quantum-dot single photon sources for quantum information technologies: A broad spectrum overview. *Appl. Phys. Rev.* **7**, 021309 (2020).

[48] Liu, J. *et al.* A solid-state source of strongly entangled photon pairs with high brightness and indistinguishability. *Nat. Nanotechnol.* **14**, 586–593 (2019).

[49] Rota, M. B. *et al.* A source of entangled photons based on a cavity-enhanced and strain-tuned GaAs quantum dot. *eLight* **4**, 13 (2024).

[50] Slichter, C. P. *Principles of Magnetic Resonance* (Springer, 1990).

[51] Mehring, M. *Principles of High Resolution NMR in Solids* (Springer Berlin Heidelberg, 1983).

[52] Burum, D. P. & Rhim, W. K. Analysis of multiple pulse NMR in solids. III. *J. Chem. Phys.* **71**, 944–956 (1979).

[53] Haeberlen, U. & Waugh, J. S. Coherent averaging effects in magnetic resonance. *Phys. Rev.* **175**, 453–467 (1968).

[54] Cowan, B. *Nuclear Magnetic Resonance and Relaxation* (Cambridge University Press, 1997).

[55] Van Vleck, J. H. The dipolar broadening of magnetic resonance lines in crystals. *Phys. Rev.* **74**, 1168–1183 (1948).

[56] Johansson, J., Nation, P. & Nori, F. Qutip 2: A Python framework for the dynamics of open quantum systems. *Comput. Phys. Commun.* **184**, 1234–1240 (2013).

[57] Taylor, J. M., Marcus, C. M. & Lukin, M. D. Long-lived memory for mesoscopic quantum bits. *Phys. Rev. Lett.* **90**, 206803 (2003).

[58] Witzel, W. M. & Das Sarma, S. Quantum theory for electron spin decoherence induced by nuclear spin dynamics in semiconductor quantum computer architectures: Spectral diffusion of localized electron spins in the nuclear solid-state environment. *Phys. Rev. B* **74**, 035322 (2006).

[59] Yang, W. & Liu, R.-B. Quantum many-body theory of qubit decoherence in a finite-size spin bath. *Phys. Rev. B* **78**, 085315 (2008).
